# Strategies to Enhance Retention in a Cohort Study Among Adults of Turkish Descent Living in Berlin

**DOI:** 10.1007/s10903-021-01309-1

**Published:** 2021-11-19

**Authors:** Christina Dornquast, Thomas Reinhold, Saliha Solak, Melike Durak, Heiko Becher, Burgi Riens, Katja Icke, Ina Danquah, Stefan N. Willich, Thomas Keil, Lilian Krist

**Affiliations:** 1 Institute of Social Medicine, Epidemiology and Health Economics, Charité – Universitätsmedizin Berlin, corporate member of Freie Universität Berlin, Humboldt-Universität Zu Berlin, and Berlin Institute of Health, Luisenstrasse 57, 10117 Berlin, Germany; 2 Institute for Medical Biometry and Epidemiology, University Medical Center Hamburg-Eppendorf, Hamburg, Germany; 3 Institute of Global Health (HIGH), Heidelberg University Hospital, Heidelberg, Germany; 4 Institute for Clinical Epidemiology and Biometry, University of Würzburg, Würzburg, Germany; 5 State Institute of Health, Bavarian Health and Food Safety Authority, Bad Kissingen, Germany

**Keywords:** Retention strategies, Cohort studies, Migrants, Participation

## Abstract

Retention is important for statistical power and external validity in long-term cohort studies. The aims of our study were to evaluate different retention strategies within a cohort study of adults of Turkish descent in Berlin, Germany, and to compare participants and non-participants. In 2011–2012, a population-based study was conducted among adults of Turkish descent to primarily examine recruitment strategies. 6 years later, the participants were re-contacted and invited to complete a self-report questionnaire regarding their health status, health care utilization, and satisfaction with medical services. The retention strategy comprised letters in both German and Turkish, phone calls, and home visits (by bilingual staff). We calculated the response rate and retention rate, using definitions of the American Association for Public Opinion Research, as well as the relative retention rate for each level of contact. Associations of baseline recruitment strategy, sociodemographic, migration-related and health-related factors with retention were investigated by logistic regression analysis. Of 557 persons contacted, 249 (44.7%) completed the questionnaire. This was 50.1% of those whose contact information was available. The relative retention rate was lowest for phone calls (8.9%) and highest for home visits (18.4%). Participants were more often non-smokers and German citizens than non-participants. For all remaining factors, no association with retention was found. In this study, among adults of Turkish descent, the retention rate increased considerably with every additional level of contact. Implementation of comprehensive retention strategies provided by culturally matched study personnel may lead to higher validity and statistical power in studies on migrant health issues.

**Supplementary Information:**

The online version contains supplementary material available at 10.1007/s10903-021-01309-1.

## Introduction

In longitudinal studies, both the initial response rate and the retention rate are crucial. Loss to follow-up may reduce external validity and the power for statistical analyses [1, 2]. The selection of which retention strategies are applied in a cohort study needs to be carefully considered. Systematic reviews showed that common retention strategies comprised reminder strategies (e.g., written reminders, phone calls, home visits, incentives), barrier-reduction strategies (e.g., childcare services, multilingual study material), community building strategies (e.g., study branding, merchandise) and tracing strategies (e.g., alternative contact persons) [3–7]. Reminder strategies followed by barrier-reduction strategies have been most frequently used in longitudinal cohort studies [4, 6]. Studies applying any barrier-reduction strategy had higher retention rates than those without [6]. Increased retention rates were mostly shown for home visits compared to other strategies [4, 8–10]. Further studies and reviews showed rather heterogeneous results regarding the usefulness of reminders and cumulative strategies [4, 6, 7, 11]. In general, active methods with personal contact (phone calls, home visits) seem to perform better than passive methods (invitation letter) [7].

The strategies presented so far have mainly been used in the context of general cohort studies. However, to obtain insights into specific groups, it is increasingly important to study ethnic minority populations as well. Due to aspects such as higher mobility or work instability, this kind of research is often more delicate and logistically difficult and frequently yields poor response rates [5]. The effectiveness of different retention steps has only scarcely been investigated for migrant populations. It is generally known that recruitment by social networks, proximity of the researchers with the community and multilingual material and study staff seemed to increase the chance of participation [5, 12–14].

Low response rates can lead to selection bias when the characteristics of participants and non-participants differ [15]. In addition to a general decline in study participation in recent decades, particular factors seem to be related to poor participation rates [16, 17]. Sociodemographic and cultural factors, such as being unmarried or living in a single-person household, having a poor education and/or low income, having a different native language or not being born in the host country, can lead to lower study participation [18–20]. Chronic diseases, poor general health, and unhealthy lifestyles have a negative impact on study participation [21, 22].

There have been only a few epidemiological studies describing reasons for non-participation over a long follow-up time. Drivsholm et al. reported a better health status among participants and higher mortality among non-participants over 20 years of follow-up in a Danish cohort study. In addition, retention was associated with higher education, not living alone, and being employed [23]. In a Canadian longitudinal pregnancy cohort, non-participants were more often younger, unmarried, had lower household income, had less education and more often identified themselves as being part of an ethnic minority [24].

While many studies described reasons for non-participation in population-based studies, there have been only a restricted number of migrant cohorts; hence, little is known about characteristics of non-participants in such cohorts [25]. Persons of Turkish descent represent the largest group of persons with migration backgrounds in Germany and, thus, they are an important target group [26]. Therefore, the aim of the present analysis was to investigate the success of different retention strategies with regard to participation among different subgroups and to compare characteristics of participants and non-participants within a cohort study among adults of Turkish descent in Berlin, Germany.

## Methods

### Study Population and Design

The present study was a cohort study among adults of Turkish descent living in Berlin. The baseline assessment took place between 2011 and 2012 in the context of the pre-test and feasibility phase of the German National Cohort Study (NAKO), which aimed to evaluate different recruitment strategies (a register-based approach and a community-oriented strategy) among Berliners of Turkish descent. A detailed description of the recruitment is published elsewhere [13, 27]. Participants received self-report questionnaires and medical examinations (height and weight, blood sample, blood pressure). The follow-up was conducted between May 2018 and July 2019. It consisted of a self-administered questionnaire asking about health status, health care utilization and satisfaction with the German health care system.

### Retention Strategies

Details of the recruitment strategy for the baseline assessment were previously published [13]. All participants who agreed at baseline to be re-contacted were invited via postal mail to participate in a follow-up assessment in 2018. The invitation letter included information about the study, a consent form, a paper-and-pencil questionnaire, a paid reply envelope and a link to an online version of the questionnaire. At each level of contact, a 15 Euro voucher for a supermarket was offered as an incentive for the completion of the questionnaire. All documents were provided in German and Turkish languages. If a potential participant had not responded within 3 weeks, he or she was contacted by phone at three different time points and different weekdays by bilingual study staff. If no contact resulted or no phone number was available, a reminder letter was sent out, followed by another reminder letter after another 3 weeks. In case this was unsuccessful, bilingual study staff conducted up to two home visits after 3 months. Participants who could be contacted at home were offered a voucher worth 30 Euros (increase in incentive approved by ethics committee). If the potential participant was not reached at home, the study team left an information card with contact details. If an invitation letter was undeliverable and/or the participant was not reachable via phone and e-mail, address searches were accomplished via registration office and post services. A flow chart of the process for all levels of contact is presented in Supplementary Fig. S1. The study was approved by the ethical review committee of the Charité-Universitätsmedizin Berlin, Germany and registered at the German Clinical Trials Register under the registration number DRKS00013545. Written informed consent was obtained from all participants.

### Participation

Participation in our study was assessed by using “response rate 1” (RR1) as the response rate and “cooperation rate 3” (COOP3) as the retention rate according to the standard definitions of the American Association for Public Opinion Research (AAPOR) [28]. Thus, RR1 was defined as the number of participants with completed follow-up questionnaires (numerator) divided by the number of participants at the first assessment point in 2011 who agreed to participate in a follow-up (denominator; including respondents, non-respondents and all cases of unknown eligibility). For calculation of the COOP3, the numerator remains the same, and the denominator was reduced by the cases of unknown eligibility (participants with no valid address), which was defined as neutral non-response. Following previously published research, we additionally defined the percentage of remaining potential participants gained at each level of contact as the relative retention rate [9].

### Questionnaire Administration

We collected data for the present study by questionnaire. For all eligible persons, it was possible to complete the questionnaire on paper or online and either in the German or Turkish language. In addition, the participants could choose between a self-administered or interviewer-based form, which was especially relevant for home visits. The questionnaire comprised sections on sociodemographic information, lifestyle, quality of life, major diseases and experiences with the health care system (utilization, satisfaction, and access barriers). Every person who actively refused to take part in the present study was asked about the main reason for his/her refusal. Potential participants with whom we were not able to make contact were defined as passive refusals.

For all potential participants (including those who had moved), general characteristics (e.g., age, sex, education) were available from the baseline assessment in 2011.

### Baseline Variables

#### Sociodemographic Factors

We included sex (male/female), age (in years), marital status (married/not married) and educational level, defined as years of attained formal education in Turkey and/or Germany (< 10 years, 10–12 years, > 12 years), as the sociodemographic variables. All participants reported their education achieved in Germany and/or Turkey.

#### Migration-related Factors

The group of migration-related variables included citizenship, mother tongue and having their own migration experience. We dichotomized the citizenship variable into German (for participants with German and Turkish or German alone citizenship) or Turkish (for participants with only Turkish citizenship). The mother tongue of the participants was assessed using a question that allowed multiple answers. We dichotomized the answers into German (alone or among others) and Turkish or other languages (except German). Migration experience was ascertained using information about the country of birth and the question of whether participants had lived in Germany since birth. People who were born in Germany and lived here since birth were defined as the group without their own migration experience. Individuals who were born in Turkey or another country were categorized into the group with their own migration experience. Also, persons who were born in Germany but did not live here since birth and migrated later are included in the group with migration experience.

#### Health-related Factors

We included variables regarding health behaviour and health status. Smoking status (assessed at baseline) was categorized into smoker (regular smoking), ex-smoker and never-smoker. Body mass index (BMI) was calculated from the body weight and height at baseline and categorized into normal weight (BMI 18.5 to < 25 kg/m^2^), overweight (BMI 25.0 to < 30 kg/m^2^), and obese (BMI ≥ 30.0 kg/m^2^). (There was no participant with a BMI below 18.5 kg/m^2^.) As the third health-related variable, we considered the self-reported presence of at least one chronic disease (yes/no) diagnosed by a physician.

### Statistical Analyses

We used an explorative statistical approach rather than conducting strict hypothesis testing. Response rate, overall retention rate, relative retention by level of contact, and the comparison of participation rates after each level of contact by participant characteristics were analysed using descriptive methods of means and standard deviations for continuous data and absolute and relative frequencies for categorical data. A multivariable logistic regression analysis was conducted to investigate associations of baseline recruitment strategy, sociodemographic variables, and migration-related and health-related factors (all included exposure variables were provided by the baseline assessment) with retention (outcome). The regression analysis was performed including participants and non-participants, while cases of neutral non-response were excluded. The results of the multivariable regression analysis are presented as odds ratios (ORs) with 95% confidence intervals (CIs). The analyses were performed using IBM SPSS Statistics for Windows 24.0 (IBM Corp., Armonk, NY).

## Results

### Retention Rate and Sample Characteristics

Of 557 persons invited, 249 participated in the follow-up, while 308 persons did not participate due to active refusal (n = 67), passive refusal (n = 181) or neutral non-response (n = 60). The response rate was 44.7%, while the retention rate was 50.1%. For improved validity, all the following results on participation refer to the retention rate. Reasons for non-participation among the active refusers were no specific reason (n = 50), lack of time (n = 10), scepticism towards data protection (n = 3), having a chronic disease (n = 3) or jail time (n = 1). Among the participants, 62.2% were women, the mean age ± standard deviation was 50 ± 12.6 years, 51.1% had no German citizenship and 75.1% had their own migration experience.

### Retention Rates by Level of Contact

**Fig. 1:**
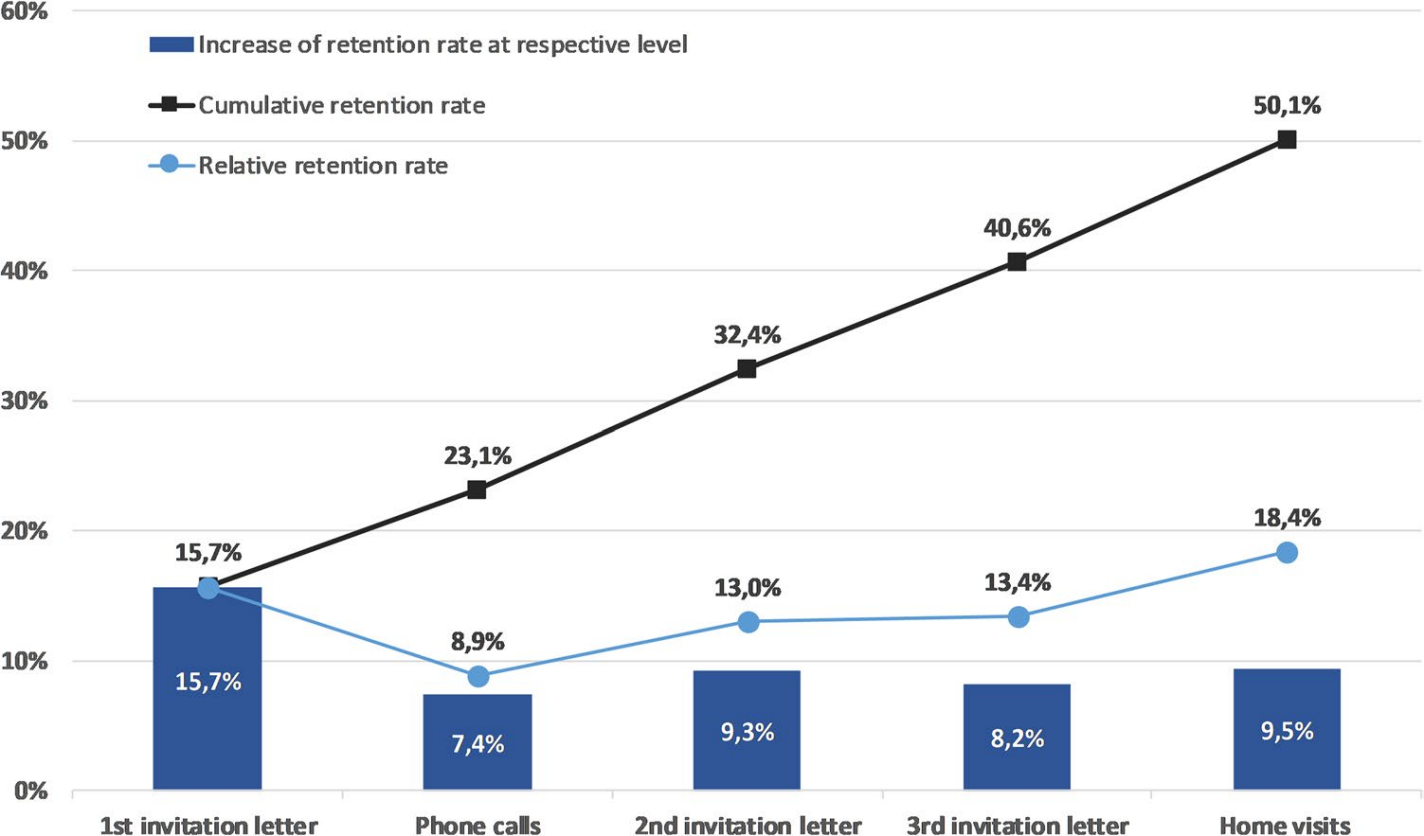
Retention rates by level of contact (Color figure online)

Each level of contact distinctly increased the retention in the present study (black line in Fig. 1). In this context, the highest increase at the respective level of contact was observed for the first invitation letter. The rise after each additional level of contact varied slightly (blue bars in Fig. 1). Regarding the relative retention rate, home visits showed the highest percentages and phone calls showed the lowest (blue line in Fig. 1).

### Reaction of Potential Participants

Regarding the different reactions of potential participants after the various levels of contact (Table 1), most persons, in absolute numbers, participated in the study after the first invitation letter. We found the highest absolute numbers of active refusals for the steps with direct contact (phone calls, home visits) made by the study staff to potential participants. In contrast, if persons reacted after one of the three written invitations, they mostly chose participation.

**Table 1:** Levels of contact

Highest level of contact	Details of recruitment process									
	Participation		No valid address found		Active refusal		Passive refusal		Total	
	n	%	n	%	n	%	n	%	n	%
First invitation letter	78	55.3	60	42.6	2	1.4	*		140	100
Phone calls	37	59.7	–		26	41.3	*		63	100
Second invitation letter	46	95.8	–		2	4.2	*		48	100
Third invitation letter	41	82.0	–		9	18.0	*		50	100
Home visits	47	18.4	–		28	10.9	181	70.7	256	100
Total	249	44.7	60	10.8	67	12.0	181	32.5	557	100

*Passive refusals at the first four levels of contact have been carried over to the next level of contact

The number of 249 participants is distributed among the individual levels of contact as follows: First invitation letter 31.3%, phone calls 14.9%, second invitation letter 18.5%, third invitation letter 16.5%, home visits 18.9%.

### Participation Among Subgroups

Among all levels of contact, participations among subgroups differed slightly. While there were only small differences between the recruitment approaches regarding age groups, more men than women compared to the total sample could be recruited via home visits. Phone calls helped to increase the response of persons without their own migration experience, and highly educated persons (Table 2).

**Table 2:** Participation among subgroups for each level of contact (percentage by column)

	First invitation letter		Phone calls		Second invitation letter		Third invitation letter		Home visits		Total sample	
	(n = 78)		(n = 37)		(n = 46)		(n = 41)		(n = 47)		(n = 249)	
	n	(%)	n	(%)	n	(%)	n	(%)	n	(%)	n	(%)
Sex												
Male	27	34.6	12	32.4	21	45.7	13	31.7	20	42.6	93	37.3
Female	51	65.4	25	67.6	25	54.3	27	65.4	27	57.4	155	62.2
Missing	–	–	–	–	–	–	1	2.4	–	–	1	0.4
Age												
< = 30	5	6.4	4	10.8	2	4.3	2	4.9	3	6.4	16	6.4
31–40	16	20.5	7	18.9	2	4.3	9	22.0	8	17.0	42	16.9
41–50	20	25.6	11	29.7	14	30.4	8	19.5	13	27.7	66	26.5
51–60	19	24.4	9	24.3	16	34.8	12	29.3	14	29.8	70	28.1
> 60	18	23.1	6	16.2	12	26.1	9	22.0	9	19.1	54	21.7
Missing	–	–	–	–	–	–	1	2.4	–	–	1	0.4
Own migration experience												
Yes	62	79.5	24	64.9	37	80.4	27	65.9	37	78.7	187	75.1
No	12	15.4	12	32.4	7	15.2	10	24.4	6	12.8	47	18.9
Missing	4	5.1	1	2.7	2	4.3	4	9.8	4	8.5	15	6.0
Education												
< 10 years	31	39.7	12	32.4	16	34.8	18	43.9	20	42.6	97	39.0
10–12 years	30	38.5	12	32.4	16	34.8	14	34.1	16	34.0	88	35.3
> 12 years	10	12.8	12	32.4	10	21.7	5	12.2	5	10.6	42	16.9
Missing	7	9.0	1	2.7	4	8.7	4	9.8	6	12.8	22	8.8

### Comparison of Participants and Non-participants

Participants differed slightly from non-participants (according to the COOP3 definition). Participants were more often married, better educated, of German citizenship, and non-smokers (i.e., ex- or never smokers). In multivariable regression analysis, we found associations between participation and never smokers [OR 2.19 (95%-CI: 1.37;3.50)] and, to a certain degree, ex-smokers [1.64 (0.96; 2.78)] compared to current smokers, as well as an association between participation and German citizenship (with or without Turkish) compared to Turkish citizenship only [1.54 (0.99; 2.39)]. Persons with missing values for educational level were less likely participants compared to persons with less than 10 years of education. All sociodemographic, migration-related and health-related characteristics of participants and non-participants are shown in Table 3.

**Table 3:** Comparison of participants vs. non-participants (according to COOP3)

	Participants n (%), mean ± SD	Non-participants n (%), mean ± SD	Adjusted OR* (95% CI)
	n = 249 (50.1)	n = 248 (49.9)	n = 431 (86.7)
Recruitment strategy			
Registration office	116 (46.8)	126 (50.8)	1
Network	132 (53.2)	122 (49.2)	1.04 (0.69;1.59)
Sociodemographic factors			
Sex			
Women	155 (62.5)	150 (60.5)	1
Men	93 (37.5)	98 (39.5)	0.73 (0.46;1.14)
Mean age at baseline	44.0 ± 12.5	43.7 ± 12.8	1.0 (0.97;1.02)
Marital status			
Married	170 (71.7)	148 (63.5)	1
Not married	67 (28.3)	85 (36.5)	0.72 (0.45;1.14)
Educational level			
< 10 years	97 (39)	102 (41.1)	1
10–12 years	88 (35.3)	80 (32.3)	1.15 (0.73;1.84)
> 12 years	42 (16.9)	26 (10.5)	1.59 (0.81;3.12)
Missing	22 (8.8)	40 (16.1)	0.39 (0.17;0.92)
Migration-related factors			
Own migration experience			
Yes	187 (79.9)	181 (79.7)	1
No	47 (20.1)	46 (20.3)	0.73 (0.38;1.40)
Citizenship			
Turkish	120 (51.1)	137 (60.1)	1
German or German/Turkish	115 (48.9)	91 (39.9)	1.54 (0.99;2.39)
Mother language			
German (alone or among others)	36 (14.5)	28 (11.3)	1
Turkish or other language	197 (79.1)	199 (80.2)	0.70 (0.37;1.30)
Missing	16 (6.4)	21 (8.5)	0.71 (0.16;3.08)
Health-related factors			
Smoking behaviour			
Smoker	84 (35.0)	129 (54.0)	1
Ex-smoker	62 (25.8)	47 (19.7)	1.64 (0.96;2.78)
Never-smoker	94 (39.2)	63 (26.4)	2.19 (1.37;3.50)
BMI			
Normal weight	56 (22.7)	69 (27.9)	1
Overweight	89 (36.0)	78 (31.6)	1.53 (0.87;2.70)
Obesity	102 (41.3)	100 (40.5)	1.28 (0.72;2.25)
At least one chronic disease (diagnosed by physician)			
No	149 (60.8)	142 (58.7)	1
Yes	87 (35.5)	79 (32.6)	1.09 (0.69;1.74)
I don’t know	9 (3.7)	21 (8.7)	0.51 (0.21;1.22)

*COOP3* Cooperation rate 3 according to the American Association of public opinion research, *OR* odds ratio, *BMI* body mass index, *SD* standard deviation

*Multivariable logistic regression analysis

## Discussion

The results of our analysis showed that half of the eligible cohort participants answered our follow-up questionnaire and that every level of contact had the potential to considerably increase the retention rate. The highest numbers of participants were observed after the first invitation letter, both overall and by subgroups. In addition, home visits showed a comparable impact on retention. The multivariable regression analysis yielded higher chances of participation for never-smokers compared to smokers and persons with German or double nationality compared to persons without German nationality.

The results of our analysis complement previous research findings for both migrant and general population studies. Regarding migrant cohorts, a current study with South Asians living in the US had a retention rate of 83% after 4.8 years of follow-up (median) [29]. This proportion is distinctly higher than our retention (50.1%) or response rate (44.7%), although the period until the follow-up attempt was comparable to our study. A systematic review of cohort studies on migrants’ health also reported a broad range of retention rates [5]. The lowest retention rates were found for a diverse migrant sample in Spain (30%) [30]. In contrast, a study among Hispanic migrants in Texas [31] showed the highest rates with 95%. Compared to these results, the retention rate of our study is in the lower-middle range. A German study from 2017 reported a retention rate of 71% for women of Turkish descent after a maximum follow-up of 1 year, which was considerably lower than the 5 years in our study and therefore could be the reason for the higher retention rate [14]. This is supported by a systematic review showing a decrease in retention with longer follow-up times [5].

In our study, we used most of the retention strategies that Teague et al. suggested in their systematic review, such as incentives, reminder letters and alternative methods of data collection (e.g., interviews face-to-face or over the phone) [6]. We also applied phone calls, phone and email messages and postal invitations, which were reported as usual methods for re-contacting by a review of longitudinal studies with migrants [5]. Concerning the use of consecutive levels of contact, Haring et al. described a comparable procedure to that used in our study. The authors similarly used several postal invitations, phone calls and home visits, and each additional recruitment step increased the retention rate of their study. The most successful one was the first postal invitation [9]. The development of our retention rate was similar to that reported by Haring et al.; however, our relative retention rate at each level of contact differed. We observed the best relative retention rate for home visits, whereas Haring et al. found the first postal invitation to be most effective [9].

Participation among different subgroups after each level of contact has rarely been investigated in previous studies. Haring et al. showed that the proportion of persons with low educational levels was higher for early respondents than for intermediate and late respondents [9]. This finding is in line with our results. The authors further reported, for example, that young people were more likely to participate in the late phases of the study. However, in our study, we could not find any differences in participation by age groups or any other characteristics.

Differences between participants and non-participants have also been examined in health surveys [15, 32–34]. In several studies, poor education, low income, higher age and smoking were reported as factors contributing to lower participation [16, 21]. However, only a few studies have examined differences between participants and non-participants in longitudinal studies [7, 35]. In our study sample, smoking behaviour was one predictor for retention. This is in line with the results of a German study conducted by Haring et al., who reported a similar effect for the follow-up assessment of the SHIP study. An explanation is that smoking is often correlated with other unfavourable health behaviour including non-participation in health studies [22, 23]. As in our study sample, no differences were observed for pre-existing diseases or BMI. Other than in our study, higher age, low education, unemployment and an urban residence also predicted non-participation [9]. Citizenship of the country where the study took place may be correlated with better health literacy, including knowledge of and active engagement in the health care system [36]. This fact could explain the higher participation of persons with German citizenship compared to those with only Turkish citizenship in our sample. Kanaya et al. investigated a multicultural study sample and found that being a woman, being Pakistani, being Muslim or having a lower educational attainment were predictors for non-participation [29]. These results indicate that factors associated with study participation depend on the respective setting and cultural specifics of the population in which the study is conducted [37].

### Strengths and Limitations

To our knowledge, this is the first cohort study in Germany examining retention and possible strategies as well as differences in participants and non-participants among persons of Turkish descent. A second strength was the use of a large number of retention strategies following current recommendations [5, 6, 12] as well as the investigation of their respective effect on retention. Third, we provided bilingual study material, and re-contact was performed by bilingual study staff to reduce barriers caused by language difficulties, as proposed in previous research [38].

A limitation of the study was the long follow-up period of 5–6 years. This may be the reason for having a considerable number of persons with unknown addresses. We were also not able to follow-up the non-participants by using a registry linkage since these registries are not available in Germany as they are in other countries [15, 23]. However, we successfully performed extensive address research, including mortality follow-up, to track persons who moved or died during the follow-up period. Since participants were recruited in a metropolitan area, the results may not be transferable to persons in a rural setting. Last, we used baseline characteristics to compare participants and non-participants, although this may not have been completely accurate, as some of these variables, such as smoking behaviour and BMI, could have changed over the follow-up period. However, as we were not able to administer a non-response questionnaire, this was our only option for making such a comparison.

### Implications for Future Research

Due to the success and impact of the extensive retention strategies applied in our study, we suggest the use of as many different retention approaches as possible in future cohort studies. In particular, strategies that involve personal contact should be considered. This applies for both general and migrant population cohorts. A second implication refers to the long interval between the baseline and follow-up assessments. This period should have a reasonable length and it is important to maintain contact with the participants. Sending postcards or making telephone calls between the various assessment points to remind the participants of your study and their involvement should be considered. Some migrant sub-populations may be hard to reach for participation in health studies [5, 39, 40]. Participants and non-participants in our study showed no differences regarding their mother tongue. This indicates that the use of bilingual materials and the involvement of bilingual study personnel need to be considered. In studies with more than one nationality as target group, personnel and translation costs have to be considered. A fourth implication arises from the observed lower retention among persons with foreign citizenship. A potential reason might be that foreign citizens are less informed or maybe less interested in local culture, customs, and practices, including the health care system, of the host country. Therefore, cultural values, norms, and traditions should be taken into account, and a more comprehensible explanation of the study aims should be considered. This may lead to a better knowledge about the study and, consequently, higher identification and retention rates.

## Conclusion

The results of our cohort study showed that each consecutive level of contact considerably increased the retention rate among study participants of Turkish descent. Therefore, investing in comprehensive retention strategies while considering cultural characteristics will lead to higher validity and higher statistical power in migrant cohort studies. If baseline characteristics are known, hard-to-reach subgroups could be addressed with more targeted strategies.

## Supplementary Information

Below is the link to the electronic supplementary material.

Supplementary file1 (PDF 626 KB) Flowchart—recruitment process
