# Supplementary figures and images for: Strategies to Enhance Retention in a Cohort Study Among Adults of Turkish Descent Living in Berlin
Source: J Immigr Minor Health. 2021 Nov 19;24(5):1309–17. doi: 10.1007/s10903-021-01309-1 (PMC9388466; doi:10.1007/s10903-021-01309-1)

Supplementary figure S1 Flowchart – Recruitment process

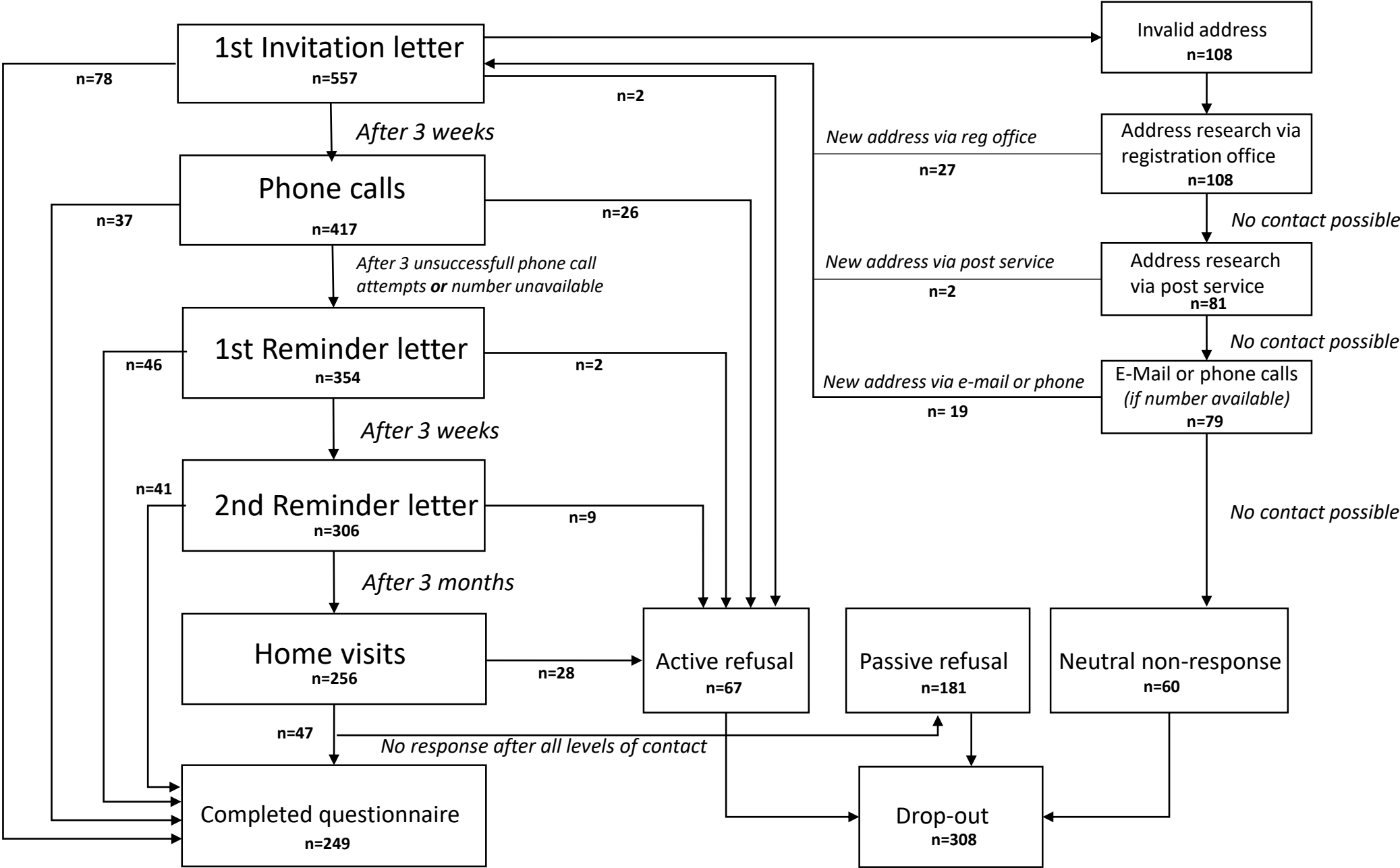

Supplement: Supplementary file 1 — Supplementary file1 (PDF 626 KB) Flowchart—recruitment process [file 10903_2021_1309_MOESM1_ESM.pdf]
